# Nudge politics: efficacy and ethics

**DOI:** 10.3389/fpsyg.2013.00972

**Published:** 2013-12-19

**Authors:** Nichola J. Raihani

**Affiliations:** Genetics, Evolution and Environment, University College LondonLondon, UK

**Keywords:** nudge, behavioral economics, ethics, libertarian paternalism

Nudge politics capitalize on psychological insights on human behavior to inform central policies. The scope for such policies to bring about large improvements in individual behavior for relatively little cost has captured the imagination of governments worldwide. “Nudging” involves using choice architecture—the ways decisions are framed or presented—to modify choosers' behavior (Thaler and Sunstein, 2008). The common assumption that people always make optimal decisions for themselves when provided with accurate information has been proven false in several contexts. Instead, people's decisions are often influenced by the context in which they are made. For example, people often overvalue immediate relative to long-term prospects, get stuck in harmful habits and are disproportionately inclined to copy the behavior of others in their social group, even to their detriment (Thaler and Sunstein, 2008). Nudges can be used to help people to overcome these tendencies. For example, they might help people save toward their pension, to choose healthier food options or to reduce their energy consumption, amongst other things (Thaler and Sunstein, 2008). Nudges are ostensibly paternalistic in that they help people to make decisions that are in their best interest (Thaler and Sunstein, 2003, 2008). At the same time, nudges are also libertarian, in that they preserve freedom of choice (Thaler and Sunstein, 2003, 2008). Here, I discuss the contexts in which nudges are likely to be most useful and also highlight some potential pitfalls with this policy-making approach. I also discuss the ethics of nudge politics, particularly when policies are designed to maximize collective, rather than individual, benefits and do not therefore fit the concept of paternalism.

In several cases, the behavior change produced by the nudge may not produce the desired policy outcome. This is especially likely when the desired outcome involves a series of repeated decisions that are made in different contexts other than that in which the nudge is implemented. Consider nudges surrounding food choices. Rising levels of obesity, and the associated population health implications, are key policy issues for governments worldwide (Just and Payne, 2009). Several nudges have been designed which can encourage healthier food choices in specific scenarios. For example, designating a larger area of the supermarket trolley for fruit and vegetables, or increasing the prominence of healthy food items in cafeterias, can encourage consumers to select more of these items (Marteau et al., 2011). However, it is currently unclear whether people who are nudged to make healthier food choices in the supermarket or the cafeteria continue to make healthier food choices in future situations, when they are not nudged. While advocates may not claim that nudges are a panacea for positive behavior change, there is a danger that some nudges may produce negative spillover effects, which risks bringing about the opposite outcome to that which was intended. For example, one study has shown that low-fat labels on food—another method used to promote healthier food choices—can actually increase caloric intake because consumers experience reduced consumption guilt (Wansink and Chandon, 2006). Nudges may be more likely to achieve the desired policy outcome when the outcome in question involves just one key decision, as is the case with encouraging participation in organ donor schemes or adding loft insulation to improve household energy efficiency. In these cases, nudges aimed at eliciting a specific decision in a specific context also simultaneously produce the desired outcome. Where outcomes are dependent on a series of decisions, policy makers should be aware that behavior change may be limited to a specific context or, worse that the nudge may produce negative spillovers, thereby negating some or all of the positive impact of the initial behavior change.

The efficacy of interventions may vary across contexts: what works well in one situation or with one group of people may be of limited use in different settings or with different cultural groups. For example, consider behavior change in the context of energy use. Data collected on over 6 million US households by the energy company, Opower, has shown that consumers can be encouraged to use less energy if they are given feedback about how their energy use compares with that of similar sized households in their neighborhood, together with tacit approval or disapproval in the form of an emoticon. These messages on the energy bill, together with suggestions of how to reduce energy use, have been shown to reduce energy consumption by around 2% (Allcott, 2011). However, the efficacy of this normative feedback varies with other factors, such as country and political ideology. Similar interventions used in the UK produced greater reductions in energy consumption than in the US (Dolan and Metcalfe, 2012, working paper), while another study showed that US households with politically liberal ideology were more likely than conservative households to reduce energy use in response to normative feedback (Costa and Kahn, 2013). These findings suggest that targeted nudges may be most effective at producing widespread behavioral change (Costa and Kahn, 2013) although such approaches are not yet widely used.

Another potential problem with nudge politics is that we don't yet know whether these interventions will work in the long term. This concern is particularly acute when nudges seek to produce outcomes which involve a series of behavioral decisions, as in the energy use example above. Reassuringly, evidence collected over a 5-year period indicates that the efficacy of this particular nudge does not attenuate; instead, consumers apparently form new consumption habits and actually reduce energy use further over time. The study does show, however, that intermittent reinforcement of the behavior with the nudge is important. In households where the treatment was stopped, energy use began to climb back up to pre-treatment levels (Allcott and Rogers, 2012, working paper).

Finally, the ethics of nudge politics are not clear cut (Hansen and Jespersen, 2013). The moral justification for nudges, according to Thaler and Sunstein (2003), rests on the assumption that they are paternalistic, or designed to make choosers better off, as judged by themselves. In reality, however, many nudges appear designed to maximize collective benefits (see Table 1 for examples). In many situations, individual and collective interests are misaligned such that producing the collective benefit requires the nudged individuals to pay upfront costs. Determining whether such nudges are morally defensible is a key challenge for policy-makers. I suggest that policy-makers may defend the use of non-paternalistic nudges if the nudged individual stands to share in the collective benefits that are produced. For example, one of the most famous examples of a successful nudge is the “presumed consent” model for organ donation. Requiring participants to opt-out, rather than opt-in, to organ donor schemes effectively doubled the number of consenting donors in one study (Johnson and Goldstein, 2003). Crucially, nudged individuals do not benefit from being nudged onto the organ donor list. Nevertheless, these individuals do share the collective benefit, in terms of increased organs available for donation which they might 1 day need.

**Table 1:** **Ethical hierarchy for policymakers when designing and implementing nudges**.

**Category of Nudge**	**Initial payoff to nudged individual**	**Collective payoff**	**Nudged individual shares collective benefit?**
**1. PATERNALISTIC**	**POSITIVE**	**POSITIVE**	**YES**
Example: Promote healthy eating by making healthy food options more prominent in cafeterias.	Health may be improved.	Less public money spent on obesity related problems.	Reduced public spending in one area frees up money for spending in another area.
**Similar nudges designed to:**			
Improve individual health or survival, e.g., behaviors relating to smoking, alcohol use, exercise; sexual behavior; seat belt use.			
Improve personal finance, e.g., join pension saving plans, on-time credit card repayments; help jobseekers find work; implement energy savings.			
**2. NON-PATERNALISTIC—TYPE A**	**NEGATIVE (OR NO COST)**	**POSITIVE**	**YES**
Example: Reduce antibiotic use by encouraging GPs to prescribe alternatives for minor ailments.	Increased risk of serious infection	Reduced emergence and spread of antibiotic-resistant pathogens.	Less likely to be infected with an antibiotic-resistant strain of infection.
**Similar nudges designed to:**			
Improve population health, e.g., increased vaccination uptake, increased participation in organ donor schemes.			
Increase tax compliance.			
Encourage costly environmental protection behavior, e.g., reusing hotel towels, reducing littering, increasing recycling.			
Increase employee effort.			
**3. NON-PATERNALISTIC—TYPE B**	**NEGATIVE**	**POSITIVE**	**NO**
Example: Increase charitable donations by enrolling employees into an opt-out scheme of donations.	Financial cost.	Recipients benefit from the donation.	Individual that donates does not share in the benefits.

Policymakers should consider the payoffs of behavior change that accrue to nudged individuals and to others that are also affected. The currencies of the payoffs may vary according to the nudge in question, for example financial nudges produce monetary payoffs whereas health nudges produce payoffs that may be estimated in terms of risk of illness or disease. Paternalistic nudges create the least challenging ethical dilemmas for policymakers, although some non-paternalistic nudges which create collective benefits that also accrue to the nudged individual (type A) may also be morally defensible. Non-paternalistic nudges which require an individual to pay a cost but share in none of the benefits (type B) are morally dubious and should be used with caution.

Nudges which require individuals to pay upfront costs without the opportunity to share in the collective benefit are morally dubious. For example, the UK Behavioural Insights Team has suggested that individuals could be nudged into more regular and generous charitable donations by using opt-out models to automatically enrol staff into workplace giving schemes (UK Behavioural Insights Team, 2013). While the aim of nudges to increase charitable donations is clearly laudable, we should think carefully before endorsing interventions which impose costs on one set of individuals (the donors) to provide benefits to another set of individuals (the recipients). Implicit in the acceptance of such an arrangement is the notion that it is morally defensible to prioritize the welfare of one group of individuals over the welfare of another group. While such a stance may be acceptable from a moral perspective under certain conditions (for example, where the benefit of receiving aid to the recipient group far outweighs the cost to the donors) it is crucial for policy-makers to stipulate precisely when this is the case.

To conclude, nudge policies have the potential to generate improvements in individuals' health, wealth and happiness for relatively small financial investments. I have highlighted a few areas where policy-makers should exercise caution, however. Specifically, the effect of nudges on behavior may be context specific. As such, policy-makers should be careful not to equate an observed change in behavior in one context with the desired policy outcome, which may depend on behavior change in several disparate contexts. In a similar vein, it would also be useful to know more about the long-term effects of behavioral interventions. On an ethical note, I argue that nudges which produce collective, rather than individual, benefits do not properly fit the concept of paternalism. Nevertheless, such nudges may often be morally defensible if the nudged individual can share in the collective benefits produced.
